# Use of Web 2.0 Social Media Platforms to Promote Community-Engaged Research Dialogs: A Preliminary Program Evaluation

**DOI:** 10.2196/resprot.4808

**Published:** 2016-09-09

**Authors:** Miguel Valdez Soto, Joyce E Balls-Berry, Shawn G Bishop, Lee A Aase, Farris K Timimi, Victor M Montori, Christi A Patten

**Affiliations:** ^1^ Center for Clinical and Translational Science Office for Community Engagement in Research Mayo Clinic Rochester, MN United States; ^2^ Department of Health Sciences Research Division of Epidemiology Mayo Clinic Rochester, MN United States; ^3^ Public Affairs Social and Digital Innovation Unit Mayo Clinic Rochester, MN United States; ^4^ Public Affairs Communications Leadership Unit Mayo Clinic Rochester, MN United States; ^5^ Mayo Clinic Social Media Network Mayo Clinic Rochester, MN United States; ^6^ Department of Cardiovascular Disease Mayo Clinic College of Medicine Rochester, MN United States; ^7^ Department of Internal Medicine Division of Endocrinology Mayo Clinic College of Medicine Rochester, MN United States; ^8^ Center for Clinical and Translational Science Knowledge and Evaluation Research Unit Mayo Clinic Rochester, MN United States; ^9^ Department of Psychiatry and Psychology Behavioral Health Research Program Mayo Clinic Rochester, MN United States

**Keywords:** Web 2.0, social media, platforms, analytics, community, engagement, stakeholders, WordPress, Twitter, Facebook

## Abstract

**Background:**

Community-engaged research is defined by the Institute of Medicine as the process of working collaboratively with groups of people affiliated by geographic proximity, special interests, or similar situations with respect to issues affecting their well-being. Traditional face-to-face community-engaged research is limited by geographic location, limited in resources, and/or uses one-way communications. Web 2.0 technologies including social media are novel communication channels for community-engaged research because these tools can reach a broader audience while promoting bidirectional dialogs.

**Objective:**

This paper reports on a preliminary program evaluation of the use of social media platforms for promoting engagement of researchers and community representatives in dialogs about community-engaged research.

**Methods:**

For this pilot program evaluation, the Clinical and Translational Science Office for Community Engagement in Research partnered with the Social Media Network at our institution to create a WordPress blog and Twitter account. Both social media platforms were facilitated by a social media manager. We used descriptive analytics for measuring engagement with WordPress and Twitter over an 18-month implementation period during 2014-2016. For the blog, we examined type of user (researcher, community representative, other) and used content analysis to generate the major themes from blog postings. For use of Twitter, we examined selected demographics and impressions among followers.

**Results:**

There were 76 blog postings observed from researchers (48/76, 64%), community representatives (23/76, 32%) and funders (5/76, 8%). The predominant themes of the blog content were research awareness and dissemination of community-engaged research (35/76, 46%) and best practices (23/76, 30%). For Twitter, we obtained 411 followers at the end of the 18-month evaluation period, with an increase of 42% (from 280 to 411) over the final 6 months. Followers reported varied geographic location (321/411, 78%, resided in the United States); 99% (407/411) spoke English; and about half (218/411, 53%) were female. Followers produced 132,000 Twitter impressions.

**Conclusions:**

Researchers and community stakeholders use social medial platforms for dialogs related to community-engaged research. This preliminary work is novel because we used Web 2.0 social media platforms to engage these stakeholders whereas prior work used face-to-face formats. Future research is needed to explore additional social media platforms; expanded reach to other diverse stakeholders including patients, providers, and payers; and additional outcomes related to engagement.

## Introduction

### Community Engagement

A 2013 Institute of Medicine report [1] report highlighted the need to promote the engagement of diverse patients, community representatives, and other stakeholders as active partners in the full spectrum of translational research. Community engagement expands research beyond the scientist-participant context by creating opportunities for meaningful, collaborative, trusting partnerships with researchers and diverse members of the community including but not limited to study participants. Community-engaged research is defined as “the process of working collaboratively with groups of people who are affiliated by geographic proximity, special interests, or similar situations with respect to issues affecting their well-being” [2]. This definition of community engagement serves as the starting place to consider novel ways to engage those interested in biomedical research. Traditional community engagement involves face-to-face outreach such as attendance at health fairs [2]. This process is restricted by geographic location and limited resources and is often characterized as a one-way communication channel. Recent approaches such as science cafes and engagement studios promote bidirectional dialogs between researchers and community members but are limited to face-to-face communication formats [3-5]. These formats promote engagement by facilitating dialogs about health needs of communities, and in turn, researchers bring perspectives on current work that addresses these needs (ie, colearning). However, community engagement biomedical research teams need new communication methods to reach and engage a larger audience. Virtual online communities could also be developed and fostered to promote community engagement [1,6-8]. This paper reports on the preliminary evaluation of the use of social media platforms for promoting engagement of researchers and community representatives in bidirectional dialogs. This work is innovative for engaging community members and researchers in dialogs using online social media platforms and has not been done previously.

### Web 2.0 Technologies

Web 2.0 transformed health communication patterns. Web 2.0 refers to a collection of electronic, Web-based applications and technologies that “facilitate interactive information sharing, user-centered design and collaboration” [9]. Web 2.0 technologies encompass a large class of information and technological tools, including blogs and social networking sites. Web 1.0 Internet-based technologies are limited to the passive viewing of content created by others [9]. In contrast, through Web 2.0 technologies users can interact and collaborate with each other in a social media dialog as cocreators of user-generated content in a virtual community. With their high level of interactivity, Web 2.0 technologies have potential for increasing the depth and reach of engagement among stakeholders [10].

### Web 2.0 Social Media Platforms

Social media tools include blogging, microblogging, social networks, and curation [11]. For example, WordPress is a free, open-source blogging tool that allows users to create webpages. Over 60.1 million new posts with 61.5 million new comments appear each month, leading to the creation of 19.1 billion pages with 409 million views [12]. Twitter offers users a different type of blogging experience called microblogging. Twitter has 288 million active monthly users with 500 million Tweets (microblogs) posted per day in more than 33 different languages [13]. Facebook, another popular social networking tool, has 936 million active users daily with 1.44 billion active monthly users [14]. Diverse individuals use social media including racial/ethnic minorities and those aged 65 years and older [15,16]. From two community health needs assessments jointly conducted by Mayo Clinic and public health partners in 2014 [17] and 2016 [18] we learned that community members prefer to receive information about health and research through social media platforms **.** In addition, social media platforms such as Twitter are used by researchers. As early as 2007, 77% of life scientists reported they used social media and, of these, 85% said these communications impacted their decision making [19,20].

### Objective

The Center for Clinical and Translational Science’s Office for Community Engagement in Research partnered with the Social Media Network at Mayo Clinic to develop and implement a social media communication plan to promote community engagement at our institution. The initial target stakeholder audiences for the social media communication plan were researchers and community representatives. Consistent with the Institute of Medicine report [1], we wanted to support and promote the use of community engagement by the workforce (ie, researchers). In addition, we targeted community representatives to increase public support for research to improve population health. For this pilot program evaluation, we created and evaluated the use of two social media platforms, Twitter and a WordPress blog, for engaging researchers and community representatives in online dialogs and community engagement educational curricula. If these platforms showed promise, our long-term goal was to develop an extensive social media plan with additional applications (eg, Facebook, Storify, and podcasts) [14,21,22], expanded targeted stakeholder audiences (eg, providers, payers, policy makers) [1,15], and engagement outcomes. This paper describes a preliminary program evaluation of the use of social media tools to engage researchers and community representative stakeholders.

## Methods

### Target Audience/Stakeholders

The audience or stakeholders targeted in this pilot program evaluation were researchers and community representatives (ie, the public). Researchers were targeted broadly along the full spectrum of clinical and translational science.

### Developing the Social Media Platforms

A key feature of our social media plan involved ensuring ease-of-use of the social media tools we selected. We took into account the Centers for Disease Control and Prevention recommendations for developing health communications [23] and specifically for engaging stakeholders through social media platforms [24] that suggested blogs and Facebook for our target audiences. We developed a WordPress blog but chose to also use Twitter based on our Mayo Clinic Social Media Network’s experience with engaging providers, researchers, and patients at our institution on health topics through this platform [15]. Blogs and Twitter forums have been successful elsewhere for connecting patients, physicians and other healthcare providers, patient and family advocacy groups, and researchers to discuss topics of interests [25].

The social media integration framework [26] provided the conceptual basis for developing the blog and Twitter social media platforms. Based on this conceptual framework, social media changes the traditional communication process through (1) exposure, (2) feedback, (3) connection, and (4) exchange. *Exposure* involves providing information to users—a blog posting by a researcher about an upcoming community outreach event, for example. While exposure begins the process of engagement, it is limited to single one-way sources of communication typical of Web 1.0 technologies. *Feedback* involves two-way communication such as a community representative responding or commenting on a researcher’s blog posting based on past experiences, opinions, and perceptions. *Connection* involves new users dialoging with one another (ie, third parties) through, for example, tweets and retweets. Finally, *exchange* involves sharing through posting of pictures, stories, testimonials, videos, podcasts, and other forms of media based on user or consumer-generated content. Sharing through stories or testimonies has been found to enhance emotional engagement and attentional focus of social media users [8]. The processes of connection and exchange promote sustainability of social media platforms [26]. Using this conceptual framework, the information flowing is not limited to one way in which stakeholders only receive messages but instead is an interactive process that places stakeholders in the center on an equal level of information exchange. This allows opportunities for bidirectional dialogs or two-way communication for community engagement using virtual communities.

### WordPress Blog

We created a WordPress blog in the spring of 2014 to increase our Web presence allowing for dynamic interactions between researchers and stakeholders [27]. The blog serves as an information hub (exposure) to explain how we engage the community in research. It allows us to archive posts in an easily accessed way for our audiences. At least two blogs are posted per week.

In addition to traditional blog posts allowing for exposure (information flow), feedback, and connecting, the WordPress site contains opportunities for sharing via video testimonials and podcasts, links to Mayo Clinic research resources, and information on how to register for educational training opportunities. Testimonials were provided by researchers conducting community-engaged research at Mayo Clinic and elsewhere. Other testimonials were sought such as a community research partner explaining the benefits of participating in research. The links to Mayo Clinic resources related directly to connecting Mayo Clinic investigators with community-engaged research liaisons available to provide mentoring and support to help study teams increase their level of engagement. Another resource available through the blog is access to the Community Engagement in Research Advisory Board. This community advisory is made of 15 community members and seeks to ensure that research conducted by and with Mayo Clinic fits the needs of the larger community. The blog also serves as portal to online educational opportunities for researchers and community to increase awareness on the principles and best practices of community engagement.

### Twitter

Twitter is an online social networking service that enables users to send and read short 140-character messages called Tweets [13]. Users can read and post Tweets. Twitter involves all of the processes by which social media can increase communication including exposure, feedback, connecting and sharing. We created a Twitter account (@mayoclinic_cenr) in August 2014 to connect researchers and stakeholders. Our research staff does purposeful microblogging to raise awareness of community-engaged research. Our staff uses the account to do live Tweets from conferences and community events. We also participate in Twitter chats hosted by other stakeholders and share newly published research and other resources related to community-engaged research.

### Social Media Facilitation

The research team developed and implemented a plan for WordPress blog postings and Tweets and identified a social media manager to facilitate the plan. The facilitator is bilingual (Spanish and English) and has a degree in business. This individual received training in social media development, facilitation, and analytics by the Mayo Clinic Social Media Network. The facilitator worked with the Network, other faculty, and staff to develop social media guidelines consistent with the overall social media plan at Mayo Clinic [15]. Guidelines for blog posts and Tweets are as follows: (1) post new blogs and Tweets at least once per week, (2) when attending a live event Tweet during the event to show active engagement, (3) have planned Tweets to send in the morning related to providing information to the followers on community engagement activities, (4) create a hashtag for the use of all social media activities (#EngagementTheNorm), (6) review and repost content from other community engagement stakeholders, and (7) stay connected with internal stakeholders relating to their needs on community-engaged research to provide updates on our research activities to the broader community. Other members of our research team posted Tweets and Blog posts as appropriate.

### Content Selection

Content for the blog and Twitter related directly to the overall capacity-building goals of our research program. These goals include increase awareness and sharing of community-engaged and collaborative research activities, develop best practices on community-engaged research in biomedical research, encourage community members and stakeholders to participate in online training on community engagement, and provide opportunities to connect around topics of shared interests to make community engagement the norm in biomedical research. These areas remained in the fore when determining the most appropriate content to share. We connected with existing collaborative partners (researchers and community members) to share information from their social media platforms and newsletters on topics of interests to our followers. This level of dynamic community engagement with other collaborative partners generated more traffic to our social media platforms.

### Promotion of the Social Media Platforms

The WordPress blog and Twitter account were promoted through the Office of Community-Engaged Research website, Mayo Clinic Public Affairs website for our Clinical and Translational Service Award, Mayo Clinic Social Media Network, colleagues from the community, and peers at other academic-medical centers.

### Evaluation Framework

**Figure 1:**
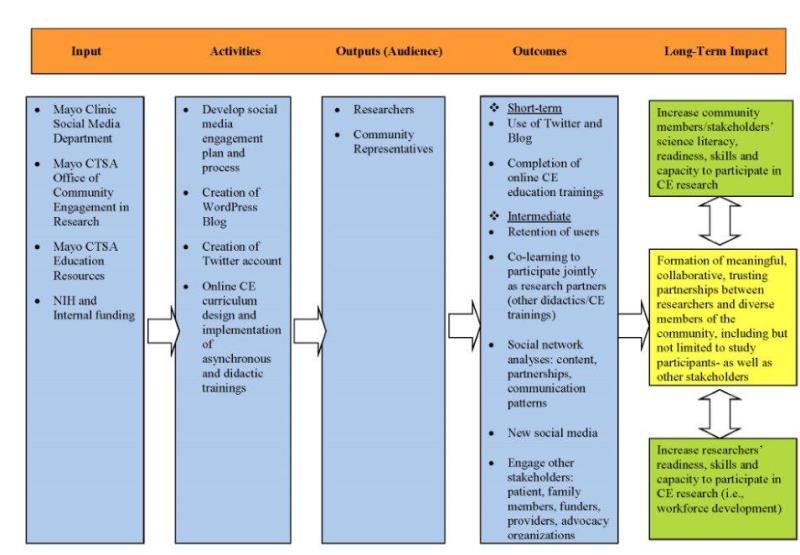
Evaluation framework.

In this preliminary evaluation we sought to summarize the use of our social media platforms as the initial step in evaluating engagement of our targeted audience. Figure 1 presents a logic model for the potential impact of social media platforms on engagement, including short, intermediate, and long-term outcomes. Proximate outcomes include use of Twitter and the blog and enrollment in our online community engagement educational curriculum among both stakeholder audiences targeted. Intermediate outcomes include increasing skills among both community representatives and researchers to build capacity for jointly conducting community-engaged research as equal partners [1]. Long-term outcomes include formation of meaningful, collaborative and trusting partnerships between researchers and diverse members of the community, including but not limited to study participants.

For this initial pilot program evaluation, a number of descriptive analytics were implemented to summarize the use of our social media platforms. Each was evaluated over an 18-month period (2014-2016). We used blog post tracking to assess use of the WordPress blog and type of user (community representative, researcher, or other). Content analysis [28] was used to generate the major themes from blog postings. Coding was done jointly by authors MVS and JBB until consensus was reached.

We used standard Twitter Analytics to determine use of this social media platform [13]. Basic Twitter Analytics are free and are linked and downloaded directly from an established Twitter account. Analytics downloaded were: (1) impressions (the number of times a user may see a tweet), engagements (the number of times a person interacts with a tweet), engagement rate (number of engagements divided by the number of impressions), and other methods of interactions with tweets such as retweets, replies, likes, user profile clicks, URL clicks, and hashtag clicks. A limited set of demographics was also available from Twitter Analytics. Data from Twitter Analytics was downloaded to an Excel spreadsheet. We used the data analysis software SPSS version 22 to calculate means and standard deviations (SDs) for selected Twitter activities.

## Results

### WordPress Blog

Over an 18-month period, we created 76 blog posts. Two-thirds of the posts were from researchers (48/76, 64%). Postings from the community represented 32% (23/76) and funders represented 8% (5/76) of all posts. From content analysis of blog postings, five themes emerged. Table 1 presents themes from the blog. The predominant theme was community-engaged research awareness and dissemination (35/76, 46%). This theme related to increasing knowledge about and findings from community-engaged research. Best practices posts (23/76, 30%) related to lessons learned about how to increase community engagement in research. Overall awareness of community engagement posts included information presented from other community engagement sources and the blogging community (15/76, 20%). Education and training posts (9/76, 12%) were linked to the creation of the new online curriculum on community engagement offered by Mayo Clinic. Community engagement events (9/76, 12%) increased awareness of activities that stakeholders could participate in either online (webinars) or in a local community. Fourteen video testimonials related to community-engaged research projects funded internally and by external partners were also posted on the blog, spanning multiple themes.

**Table 1:** WordPress blog themes from 76 postings over an 18-month period (2014-2016).

Blog theme	n (%)^a^	Illustrative content
Research awareness and dissemination	35 (46)	Title of post (testimonial from a community partner, see Multimedia Appendix 1): What to do and what to avoid when doing outreach.
Best practices	23 (30)	Title of post (from a researcher, see Multimedia Appendix 2): How do you address community's needs if they are different than your original project?
Overall awareness	15 (20)	Title of post (Mayo podcast [29,30], see Multimedia Appendix 3): How to navigate health care.
Education and training	9 (12)	Title of post (from a researcher, see Multimedia Appendix 4): A New Year's resolution you can accomplish!
Events	9 (12)	Title of post (from a community representative, see Multimedia Appendix 5): Mark your calendar if you want to outreach to the Latino community –Partnership with Alliance of Chicanos Hispanics and Latin Americans.

^a^Percentages do not equal 100 as some postings reflected multiple themes and categories are not exclusive.

### Twitter

At the start of the evaluation period we had no followers. Over an 18-month period, we acquired 411 followers, with one new follower added nearly daily. From 12 to 18 months, we increased the number of followers by 42% (from 240 to 411). Table 2 presents selected demographics of the 411 followers.

**Table 2:** Selected demographic characteristics of Twitter followers (N=411) over an 18-month period (2014-2016).

Characteristic		n (%)^a^
**Gender identity**		
	Female	218 (53.0)
	Male	193 (47.0)
**Language**		
	English	407 (94.2)
	Spanish	16 (3.7)
	French	3 (<1.0)
	Portuguese	3 (<1.0)
	Arabic	3 (<1.0)
**Country**		
	United States	321 (78.1)
	Canada	25 (6.1)
	United Kingdom	8 (1.9)
	Australia	8 (1.9)
	Mexico	5 (1.2)
**North American region**		
	Minnesota	103 (32.1)
	Florida	16 (5.0)
	California	13 (4.0)
	Illinois	13 (4.0)
	New York	10 (3.1)
	Ontario, CA	10 (3.1)
	Massachusetts	10 (3.1)
	Virginia	6 (1.9)
	Wisconsin	6 (1.9)
	Pennsylvania	6 (1.9)
**Top interests**		
	Business and news	292 (71.0)
	Health, mind, and body	284 (69.1)
	Politics and current events	267 (65.0)
	Science news	263 (64.0)
	Biotech and biomedical	230 (56.0)
	Tech news	230 (56.0)
	Technology	185 (45.0)
**Device type**		
	Desktop or laptop computer	325 (79.1)
	iOS device	185 (45.0)
	Android device	95 (23.1)

^a^For some categories, percentages do not equal 100 due to multiple responses being possible.

**Table 3:** Twitter use (N=411) for an 18-month period (2014-2016).

Twitter Analytics	Mean (SD)
Impressions	184.29 (734.36)
Engagements	4.05 (6.58)
Engagement rate	0.27 (0.33)
Retweets	0.42 (1.00)
Replies	0.14 (0.48)
Likes	0.58 (1.02)
User profile clicks	0.36 (0.83)
URL clicks	0.56 (1.50)
Hashtag clicks	0.15 (0.56)

Table 3 shows the level of interactions with the Twitter microblogs and our followers. Followers produced 132,000 Twitter impressions.

Twitter followers were engaging with our content with new discussions/topics generated. Although we did not systematically collect content data for Tweets, examination of some of the topic areas indicated a health focus—cancer, diabetes, blood pressure, substance use, and mental health—and information on upcoming community outreach events addressing these health topics.

### Preliminary Impact of Social Media on Engagement With Online Community Engagement in Research Curriculum

Twitter was used to promote free educational training opportunities on community-engaged research offered by our research team that were hosted on the WordPress blog. We tweeted when new trainings were available on the blog. As noted above, the blog apparently served to increase awareness of our community-engaged research educational online curriculum (Table 1). Twitter was also used to promote this free online curriculum that was offered to community members and researchers. In February 2015, we had 40 learners complete this online training. After that time, we increased promotion of these trainings by creating a Tweet pin and promoting our educational opportunities during Tweet chats. At the end of the 18-month evaluation period, our learner base more than tripled with 182 learners. Of these learners, 19 were community members and 163 were researchers.

## Discussion

### Principal Findings

Increasing attention has focused on engagement of stakeholders to enhance research translation [1,2]. This preliminary program evaluation examined if researchers and community representatives would use social media platforms to dialog and interact around community-engaged research. The main finding was that researchers and community stakeholders use social media platforms to engage in dialogs. We were able to engage with these stakeholders by posting at least two blogs per week and more frequent Tweets. A potential concern was that only researchers would use these platforms, but about one-third (23/76, 32%) of the blog postings were from community representatives. It should also be noted that from Twitter Analytics (Table 2), the main interests of our followers were business and news; health, mind, and body; and politics and current events. Prior studies used traditional forms of community engagement between researchers and stakeholders including face-to-face outreach [2-5]. These strategies are limited by available resources and geographic location. Our findings are innovative because we used social media platforms to promote discussions between researchers and community representatives with a large geographical reach. Using Twitter Analytics gave us a bit of insight on our audience of Twitter followers that might use social media platforms to dialog about community-engaged research. Of note, we attracted a geographically diverse audience from the United States and other countries which speaks to the potential reach of Web 2.0 technologies for community-engaged research. Through content analysis we explored the types of information exchange on the blog which produced novel data, particularly with respect to increasing knowledge about and findings from community-engaged research. Benefits our staff observed were that the social media platforms provided a new method for dissemination of research findings, raised awareness of scientific leaders in community-engaged research, and helped develop a core network of diverse communities communicating about health research. Our preliminary results further indicate that social media platforms can potentially impact engagement of community members and other stakeholders in online community engagement educational trainings.

### Limitations

A key limitation to our work was the use of Twitter and WordPress analytics. The demographic data are extremely limited in scope, and we are not able to examine some areas of interests and trends beyond basic awareness of our posts. In particular, we did not assess the racial/ethnic or socioeconomic characteristics of our blog users or Twitter followers. Moreover, although we were able to assess the type of blog user (eg, researcher, community representative, or other stakeholder), we did not collect these data for our Twitter followers. We did not determine what type of researchers across the full spectrum of clinical and translational science engaged with the social media. We are therefore unable to compare our samples with general population characteristics to assess representativeness. Another drawback: the WordPress blog readers did not post comments on the blog posts although this feature is available. We also made it possible for blog readers to contact us directly with feedback and suggestions. However, these features were not specifically promoted, which could have helped to increase dynamic engagement with our Web 2.0 social media platforms [31]. Information was not available on number of Twitter followers over various time intervals that would have allowed us to examine trends in the data, although we did observe a 42% increase in the number of followers over the final 6 months after creating the Twitter account. We did not assess if we retained users or long-term engagement [6]. Furthermore, we did not obtain complete data on the content of Tweets which would have provided useful information on the types of dialogs engaged in by various stakeholders. Encouragingly, a limited evaluation of some of the Tweet content generated indicated a health and wellness focus and sharing of upcoming information on community outreach events addressing health topics.

Another limitation is that we did not vary engagement activities in a clear experimental framework. Moreover, we do not have baseline data from which to compare our results. In addition, this preliminary evaluation is limited by the use of only two social media platforms, and other very popular technologies exist such as Facebook. Another drawback is that we only targeted specific stakeholders of researchers and community representatives. Moreover, resources were limited for promoting the use of the social media platforms to researchers and the community and thus their potential use may be underestimated.

### Future Directions

This work suggests several directions for future research. We plan to extend the reach of our approach by using additional social networking tools such as Facebook, Instagram, and podcasts. We will utilize innovative platforms including crowdsourcing to assess public views on research topics as a form of engagement. Moreover, we plan to use tools such as Storify.com to moderate social media–based conversations related to community-engaged research. Future research is also needed to expand the targeted stakeholder audiences to patients, providers, and payers [1,15]. To extend the reach of our social media platforms for community-engaged research, creative and targeted efforts are needed to reach racially and socioeconomically diverse stakeholders. We will develop an integrated communications plan which is essential to promoting community-engaged research [32]. Future promotions such as flyers and billboards will include a QR code linking to the social media platforms. As a preliminary evaluation, our purpose was to engage researchers and community representatives in discussions about community-engaged research. Future research might select specific populations with known sociodemographics and measure engagement with social media platforms, comparing demographics of those who use the applications versus those who do not.

Our platforms were nondirective, and certain topics or questions such as health needs among community representatives were not explored. One study of a Twitter-based intervention for smoking cessation [33] used a hybrid approach combining a traditional social media approach of spontaneous, real-time automated messages that encouraged discussions of focused topics with online community building [6,7], promoting sustainability. This approach could be evaluated in future evaluations.

Our preliminary results suggest that the potential impact of social media to promote engagement of community members and other diverse stakeholders in community engagement educational trainings needs further evaluation. We now have a baseline level of participation in our online community engagement educational curriculum using Twitter and the blog; future evaluations can test impact of different social media platforms and promotion strategies. In particular, we need to expand our efforts to promote our education and training in community-engaged research among community members.

Studies are warranted to evaluate use of social media platforms for impact on outcomes specified in Figure 1. Survey research is needed to assess retention of users and long-term engagement [6]. Furthermore, the application of social network analysis is a promising and innovative approach for assessing engagement outcomes in future work [6]. Social network analysis could be used to examine trends in the content of the social media dialogs, demonstrate relationships and connections between members (eg, influential users, patterns of communication), and identify gaps in our communication plan for reaching diverse groups of community representatives and researchers.

### Conclusion

In conclusion, researchers and community member stakeholders use social media platforms for dialogs related to community-engaged research. Moreover, social media platforms could engage these stakeholders to participate in community engagement educational trainings. Based on this preliminary program evaluation, Web 2.0 technologies hold great promise for engaging stakeholders in clinical and translational science research.

## Appendix

Multimedia Appendix 1 What to do and what to avoid when doing outreach.

Multimedia Appendix 2 How do you address community's needs if they are different than your original project?

Multimedia Appendix 3 How to navigate health care.

Multimedia Appendix 4 A New Year's resolution you can accomplish!

Multimedia Appendix 5 Mark your calendar if you want to outreach to the Latino community –Partnership with Alliance of Chicanos Hispanics and Latin Americans.

## Abbreviations

SD: standard deviation
