# Supplementary figures and images for: Use of Web 2.0 Social Media Platforms to Promote Community-Engaged Research Dialogs: A Preliminary Program Evaluation
Source: JMIR Res Protoc. 2016 Sep 9;5(3):e183. doi: 10.2196/resprot.4808 (PMC5035380; doi:10.2196/resprot.4808)

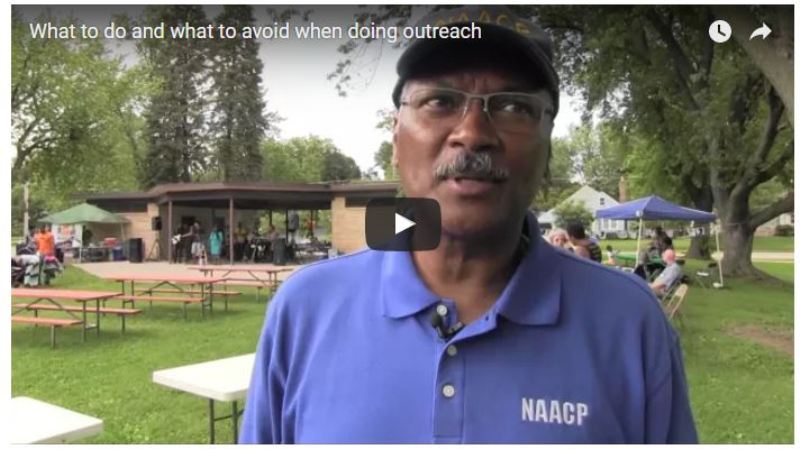

Supplement: Multimedia Appendix 1 [file resprot_v5i3e183_app1.jpg]

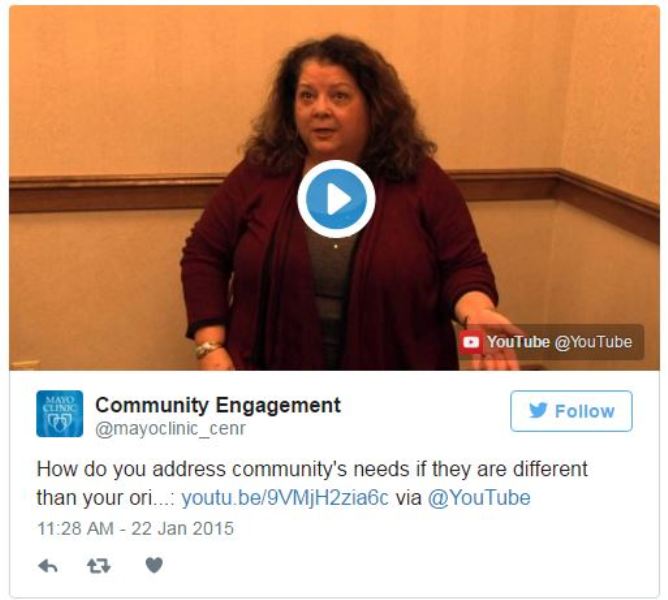

Supplement: Multimedia Appendix 2 [file resprot_v5i3e183_app2.jpg]

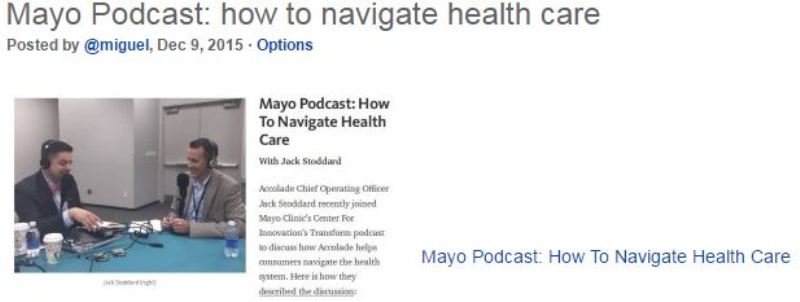

Supplement: Multimedia Appendix 3 [file resprot_v5i3e183_app3.jpg]

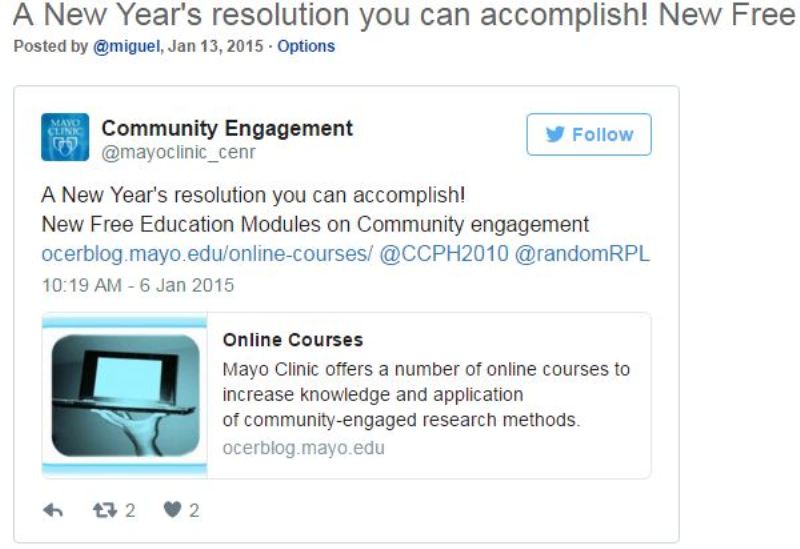

Supplement: Multimedia Appendix 4 [file resprot_v5i3e183_app4.jpg]

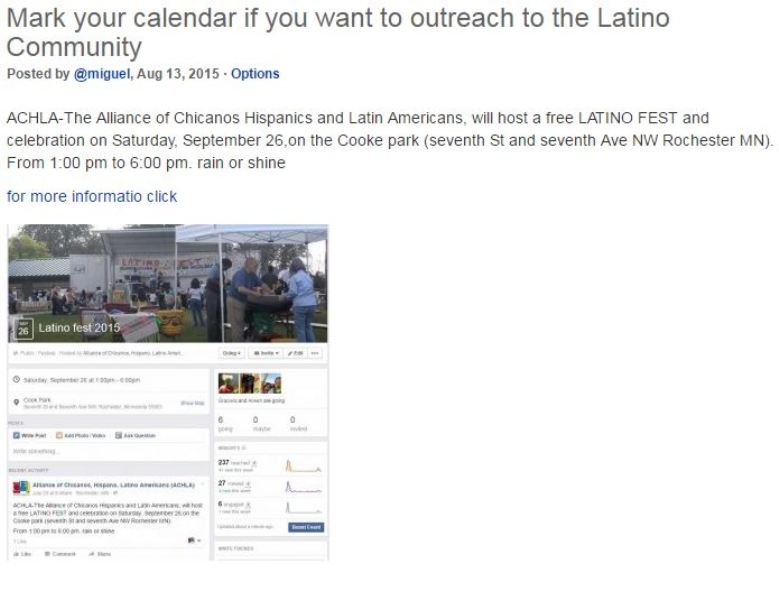

Supplement: Multimedia Appendix 5 [file resprot_v5i3e183_app5.jpg]
